# *Staphylococcus parequorum* sp. nov. and *Staphylococcus halotolerans* sp. nov., isolated from traditional Korean soybean foods

**DOI:** 10.71150/jm.2503003

**Published:** 2025-08-31

**Authors:** Ju Hye Baek, Dong Min Han, Dae Gyu Choi, Chae Yeong Moon, Jae Kyeong Lee, Chul-Hong Kim, Jung-Woong Kim, Che Ok Jeon

**Affiliations:** Department of Life Science, Chung-Ang University, Seoul 06974, Republic of Korea

**Keywords:** *Staphylococcus parequorum*, *Staphylococcus halotolerans*, new taxa, soybean foods, coagulase-negative staphylococci, antibiotic resistance

## Abstract

Strains Mo2-6^T^, S9, KG4-3^T^, and 50Mo3-2, identified as coagulase-negative, Gram-stain-positive, halotolerant, non-motile coccoid bacteria, were isolated from traditional Korean soybean foods. Strains Mo2-6^T^ and S9 were both catalase- and oxidase-negative, whereas KG4-3^T^ and 50Mo3-2 were catalase-positive but oxidase-negative. The optimal growth conditions for Mo2-6^T^ and S9 were 30°C, 2% NaCl, and pH 7.0, while KG4-3^T^ and 50Mo3-2 grew best at 35°C, 2% NaCl, and pH 7.0. All strains contained menaquinone-7 as the predominant isoprenoid quinone, with anteiso-C_15:0_ and iso-C_15:0_ as the major cellular fatty acids (> 10%). Additionally, anteiso-C_13:0_ was a major fatty acid in strain KG4-3^T^. The DNA G + C contents of strains Mo2-6^T^, S9, KG4-3^T^, and 50Mo3-2 were 33.4%, 33.3%, 32.5%, and 32.7%, respectively. Phylogenetic analyses based on the 16S rRNA gene and whole-genome sequences revealed that strains Mo2-6^T^ and S9, as well as KG4-3^T^ and 50Mo3-2, formed distinct lineages within the genus *Staphylococcus*. Digital DNA-DNA hybridization (dDDH) and average nucleotide identity (ANI) analyses confirmed that strains Mo2-6^T^ and S9, as well as KG4-3^T^ and 50Mo3-2, belonged to the same species. Meanwhile, dDDH and ANI values between strains Mo2-6^T^ and KG4-3^T^, as well as comparisons with other *Staphylococcus* type strains, were below the species delineation thresholds, indicating they represent novel species. Based on phenotypic, chemotaxonomic, and molecular data, we propose strain Mo2-6^T^ as the type strain of *Staphylococcus parequorum* sp. nov. (=KACC 23685^T^ =JCM 37038^T^) and strain KG4-3^T^ as the type strain of *Staphylococcus halotolerans* sp. nov. (=KACC 23684^T^ =JCM 37037^T^).

## Introduction

The genus *Staphylococcus*, belonging to the family *Staphylococcaceae* within the phylum Bacillota, was first described by Rosenbach (1884), with *Staphylococcus aureus* designated as the type species. As of May 29, 2025, the genus comprises 70 validly published species (Parte et al., 2020; https://lpsn.dsmz.de/genus/Staphylococcus). Members of *Staphylococcus* are typically Gram-positive, non-endospore-forming, and non-motile cocci that occur singly, in pairs, tetrads, or short chains (3–4 cells), often forming irregular, grape-like clusters (Whitman et al., 2015). Most species are facultative anaerobes, though some are strictly anaerobic. Their cell walls contain peptidoglycan and teichoic acid, and they generally possess menaquinone-7 (MK-7) as the predominant isoprenoid quinone, although some species may also contain MK-6 or MK-8. *Staphylococcus* species are widely distributed in various environments, including human clinical samples (Chew et al., 2021; Kovařovic et al., 2023; Murray et al., 2018; Pain et al., 2020; Schutte et al., 2021), animals (Kovařovic et al., 2022; MacFadyen et al., 2019; Newstead et al., 2021), plants (Cho et al., 2022), animal feces (Volokhov et al., 2023), air (Kozajda et al., 2019), and even cold environments such as Antarctica (Pantůček et al., 2018). Additionally, due to their salt tolerance, *Staphylococcus* strains are commonly found in high-salt fermented foods (Han et al., 2021; Jeong et al., 2014).

*Staphylococcus* species are broadly classified as either coagulase-positive (CoPS) or coagulase-negative (CoNS) based on their ability to produce coagulase (Becker et al., 2014). CoPS strains are well-known human pathogens associated with significant morbidity and mortality (Lee et al., 2018; Nguyen et al., 2024). In contrast, CoNS strains generally exhibit lower pathogenicity but are still considered opportunistic pathogens due to their antibiotic resistance and toxin production (Irlinger, 2008). However, in the food industry, CoNS strains isolated from fermented foods are valued as starter cultures due to their low pathogenicity and beneficial properties, including flavor enhancement and improved sensory characteristics (Khusro and Aarti, 2022; Stavropoulou and Leroy, 2016). In this study, we isolated four coagulase-negative *Staphylococcus* strains from traditional Korean soybean foods and characterized their taxonomic properties using a polyphasic approach.

## Materials and Methods

### Isolation of *Staphylococcus* strains and culture conditions

Strains Mo2-6^T^, S9, KG4-3^T^, and 50Mo3-2 were isolated from ganjang, a traditional Korean soy sauce, as previously described (Lee et al., 2024). Ganjang was prepared by soaking meju (fermented soybean bricks) in a 16% (w/v) solar salt solution (Jung et al., 2015). After 30 days of fermentation, liquid samples were collected, serially diluted in phosphate-buffered saline (137 mM NaCl, 2.7 mM KCl, 10 mM Na_2_HPO_4_, 2 mM KH_2_PO_4_, pH 7.2), and spread on tryptic soy agar (TSA; MBcell, Korea) containing 10% (w/v) NaCl. Plates were incubated at 30°C for 5 days. Genomic DNA was extracted from colonies by boiling in 5% Chelex 100 solution (Bio-Rad, USA) for 10 min. 16S rRNA genes were amplified using primers 27F and 1492R, digested with HaeIII and HhaI, and representative amplicons were partially sequenced with primer 340F (Lee et al., 2024). Sequences were compared with those of type strains using the Nucleotide Similarity Search tool (http://www.ezbiocloud.net/identify/) (Yoon et al., 2017). All four strains were identified as members of the genus *Staphylococcus* and selected for further phenotypic and phylogenetic characterization. Strains were routinely cultured on TSA with 2% (w/v) NaCl at 30°C and stored in TSB (TSB; MBcell) with 15% (v/v) glycerol at -80°C.

### 16S rRNA sequencing and phylogenetic analysis

The 16S rRNA genes of strains Mo2-6^T^, S9, KG4-3^T^, and 50Mo3-2 were initially amplified using primers 27F and 1492R and further sequenced with primers 518R and 805F (Lee et al., 2023). Nearly complete sequences were assembled from reads obtained with primers 340F, 518R, and 805F. Sequence similarities to type strains were assessed using the Nucleotide Similarity Search program. Multiple sequence alignments were performed using Infernal (v1.1.4) with the RF00177 covariance model (Nawrocki and Eddy, 2013). Phylogenetic trees were constructed using neighbor-joining (NJ), maximum-parsimony (MP), and maximum-likelihood (ML) methods, each with 1,000 bootstrap replications. The NJ tree was generated using the Kimura two-parameter model; the MP tree employed the nearest-neighbor-interchange heuristic method, and the ML tree used pairwise deletion. All analyses were performed using MEGA11 (Tamura et al., 2021).

### Whole-genome sequencing and genome-based phylogeny

Genomic DNA from strains Mo2-6^T^, S9, KG4-3^T^, and 50Mo3-2 was extracted from cultures grown in TSB with 2% NaCl using the Wizard Genomic DNA Purification Kit (Promega, USA), following the manufacturer’s instructions. Whole-genome sequencing was conducted using a hybrid approach combining the Oxford Nanopore MinION platform (ONT, UK) and the Illumina Mini-Seq platform (Illumina, USA) with 151 bp paired-end reads. Long-read data from the Nanopore MinION were de novo assembled using Flye (v2.9.1; Kolmogorov et al., 2019). The assembled long contigs were subsequently polished with Illumina short-read data using Pilon (v1.24; Walker et al., 2014), with multiple rounds of polishing conducted until no further corrections were identified. Genome completeness and contamination were assessed with CheckM2 (v1.0.2; Chklovski et al., 2023). Phylogenomic analysis was conducted using GTDB-Tk, based on concatenated protein sequences of 120 single-copy marker genes (bac120 set) (Chaumeil et al., 2020). Maximum-likelihood phylogenomic trees were reconstructed in MEGA11 with 1,000 bootstrap replications. Genomic similarity was further evaluated using average nucleotide identity (ANI) and digital DNA-DNA hybridization (dDDH). ANI values were calculated using the Orthologous ANI Tool (OAT v0.93.1; www.ezbiocloud.net/tools/orthoani) (Lee et al., 2016), and dDDH values were estimated using the Genome-to-Genome Distance Calculator (GGDC 3.0; https://ggdc.dsmz.de/ggdc.php) with formula 2 (Meier-Kolthoff et al., 2013).

### Genomic analysis

The whole-genome sequences of strains Mo2-6^T^ (CP150682–3), S9 (CP150678–81), KG4-3^T^ (CP166245–6), and 50Mo3-2 (JBBUKX000000000) were deposited in GenBank and annotated using the NCBI Prokaryotic Genome Annotation Pipeline (Tatusova et al., 2016). Antibiotic resistance genes in these strains and their closely related type strains were identified using ABRicate v1.0.1 (https://github.com/tseemann/abricate) with the CARD (Jia et al., 2017) and ResFinder (Zankari et al., 2012) databases, applying thresholds of > 70% coverage and > 50% identity. The presence of staphylococcal cassette chromosome mec (SCCmec) elements was further evaluated using the staphopia-sccmec tool (Petit and Read, 2018).

### Phenotypic and biochemical analysis

The growth of strains Mo2-6^T^, S9, KG4-3^T^, and 50Mo3-2, along with three reference strains, was assessed on various bacteriological media—including TSA, nutrient agar (NA), Reasoner’s 2A (R2A) agar, Luria-Bertani (LB) agar, and marine agar (MA) (all from MBcell)—supplemented with ~2% NaCl and incubated at 30°C for 2 days. Salt tolerance was tested in TSB with 0–30% (w/v) NaCl in 2% increments. Temperature-dependent growth was evaluated on TSA with 2% NaCl at 4, 10, 15, 20, 25, 30, 35, 40, and 45°C.

All subsequent tests were conducted on TSA or TSB supplemented with 2% NaCl at optimal temperatures. pH tolerance was assessed in TSB adjusted to pH 3.0–11.0 in 1.0-unit increments, buffered with sodium citrate (pH 3.0–5.0), sodium phosphate (pH 6.0–7.0), and sodium carbonate (pH 8.0–11.0) (Gomori, 2010). pH was readjusted after autoclaving, if necessary. Anaerobic growth was tested on TSA using the GasPak Plus system (BBL, USA) for 2 days. Gram staining was performed using a commercial kit (bioMérieux, France) according to the manufacturer’s instructions. Cell morphology and motility were examined under a phase-contrast microscope (Zeiss Axio Scope A1; Carl Zeiss, Germany) using cells grown on TSA for 2 days. Motility was further confirmed on TSA containing 0.3% (w/v) agar. For detailed morphological and flagellar observation, cells were placed on formvar-coated copper grids, negatively stained with 2% (w/v) uranyl acetate (Sigma-Aldrich, USA) for 15 s, and examined under a transmission electron microscope (JEM-1010; JEOL, Japan). Endospore formation was evaluated by malachite green staining of cells grown in TSB containing 2% NaCl and 5 mg/L MnSO_4_ at 30°C for 5 days, following the method of Smibert and Krieg (1994). Catalase activity was determined by bubble formation in a 3% (v/v) hydrogen peroxide solution, and oxidase activity was assessed using 1% (w/v) tetramethyl-*p*-phenylenediamine (Merck, USA) (Smibert and Krieg, 1994). Coagulase activity was tested with rabbit plasma (MBcell). Hydrolytic activities for casein (1% skimmed milk), starch (1%), esculin (0.1%), L-tyrosine (0.5%), Tween 20 (1%), and Tween 80 (1%) were evaluated on TSA, following Smibert and Krieg (1994). Additional biochemical and enzymatic properties of strains Mo2-6^T^, S9, KG4-3^T^, and 50Mo3-2, as well as three reference strains, were examined using API 20NE kits (bioMérieux), with cells suspended in 2% NaCl solution for inoculation.

Antimicrobial susceptibility tests were conducted on TSA supplemented with 2% NaCl and various antibiotics at the following concentrations: ampicillin (10 mg/L), penicillin (1 mg/L), erythromycin (10 mg/L), tetracycline (10 mg/L), fosfomycin (10 mg/L), streptomycin (50 mg/L), ciprofloxacin (10 mg/L), chloramphenicol (10 mg/L), kanamycin (30 mg/L), rifampicin (10 mg/L), oxacillin (10 mg/L), and vancomycin (10 mg/L). After autoclaving, antibiotics were added to cooled TSA, mixed thoroughly, and poured into plates. Each strain was suspended in 0.9% (w/v) saline and spread onto the antibiotic-containing plates at approximately 10⁴ CFU per plate. Growth was evaluated after 16–20 h of incubation, depending on the antibiotic.

### Chemotaxonomic analysis

For respiratory isoprenoid quinone analysis, strains Mo2-6^T^, S9, KG4-3^T^, and 50Mo3-2 were grown to the exponential phase in TSB supplemented with 2% NaCl at their optimal temperatures. Cells were harvested by centrifugation, and isoprenoid quinones were extracted following the method of Minnikin et al. (1984). The quinones were analyzed using an HPLC system (LC-20A; Shimadzu, Japan) equipped with a diode array detector (SPD-M20A; Shimadzu) and a reversed-phase column (250 × 4.6 mm, Kromasil; Akzo Nobel), with methanol-isopropanol (2:1, v/v) as the mobile phase at a flow rate of 1 ml/min.

For cellular fatty acid analysis, strains Mo2-6^T^, S9, KG4-3^T^, and 50Mo3-2, along with reference strains, were cultured in TSB supplemented with 2% NaCl at their optimal growth temperatures. Cells were harvested during the exponential phase (OD_600_ = 0.7–0.8), and fatty acids were saponified, methylated, and extracted following the standard MIDI protocol. Fatty acid methyl esters were analyzed using a Hewlett Packard 6890 gas chromatograph, and their composition was determined using the RTSBA6 database of the Microbial Identification System (Sherlock version 6.0B) (Sasser, 1990).

## Results and Discussion

### Isolation of *Staphylococcus* strains

*Staphylococcus* strains are known for their high salt tolerance but typically grow best at ~2% NaCl (Kong et al., 2022). Initially, we used TSA with 2% NaCl to isolate *Staphylococcus* from ganjang fermentation samples; however, the plates were dominated by other bacteria, such as *Bacillus* and *Enterococcus* (data not shown). To selectively isolate *Staphylococcus*, we used TSA with 10% NaCl, which suppressed less salt-tolerant species and enabled the successful isolation of several *Staphylococcus* strains, including Mo2-6^T^, S9, KG4-3^T^, and 50Mo3-2. These strains were isolated from different ganjang fermentation batches, and their phenotypic and phylogenetic characteristics were analyzed in this study for taxonomic classification.

### Phylogenetic analysis based on 16S rRNA gene sequences

**Fig. 1.**
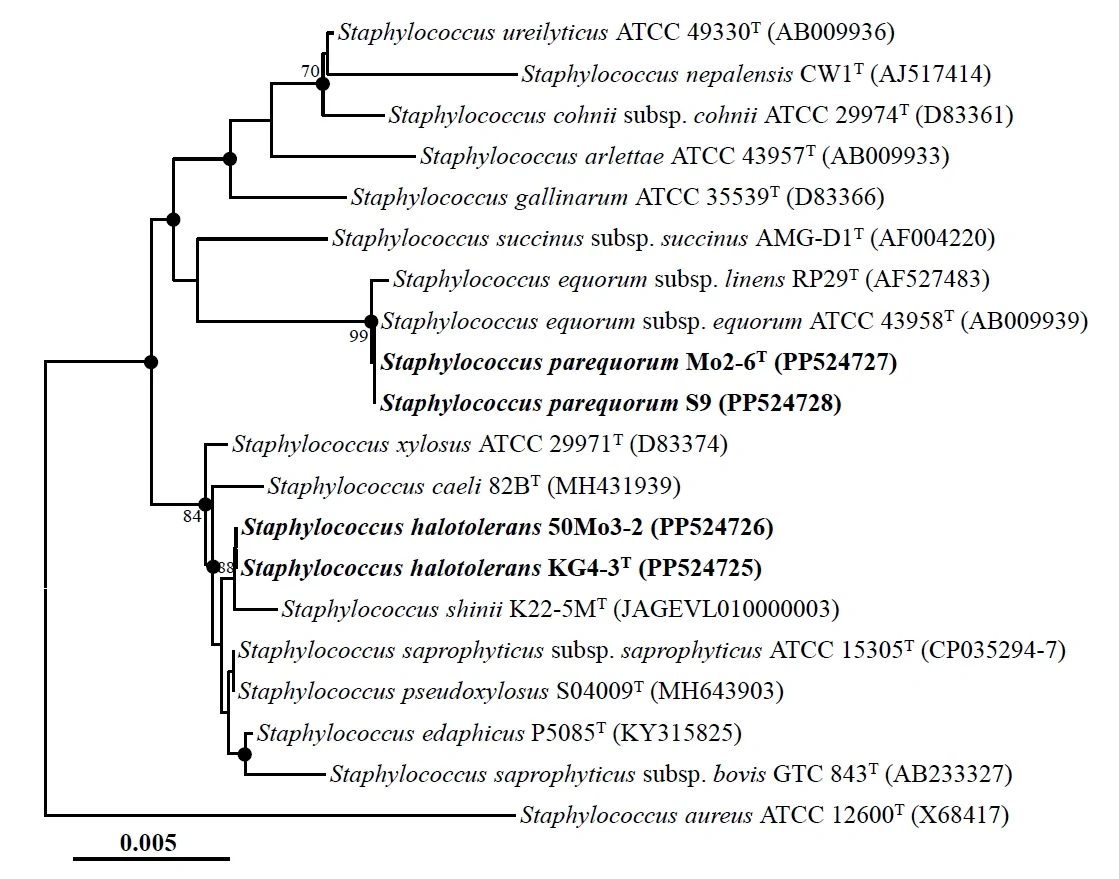
Neighbor-joining tree showing the phylogenetic relationships among strains Mo2-6^T^, S9, KG4-3^T^, and 50Mo3-2, and their closely related type strains, based on 16S rRNA gene sequences. The solid circles indicate nodes that were also supported in the maximum-likelihood and maximum-parsimony trees. The numbers at the nodes represent bootstrap percentages from 1,000 replicates; only values greater than 70% are shown. *Staphylococcus aureus* ATCC 12600^T^ (X68417) was used as the outgroup. Scale bar: 0.005 substitutions per nucleotide.

Nearly complete 16S rRNA gene sequences were obtained for strains Mo2-6^T^ (1,494 nucleotides), S9 (1,494 nucleotides), KG4-3^T^ (1,495 nucleotides), and 50Mo3-2 (1,493 nucleotides) by sequencing and assembling their 16S rRNA gene amplicons using primers 340F, 518R, and 805F. Comparative analysis of these sequences revealed high similarities: strains Mo2-6^T^ and S9 were identical (100% similarity), strains KG4-3^T^ and 50Mo3-2 shared 99.9% similarity, and strains Mo2-6^T^ and KG4-3^T^ shared 99.0% similarity. Phylogenetic analysis based on 16S rRNA gene sequences using the NJ algorithm revealed that strains Mo2-6^T^ and S9, as well as strains KG4-3^T^ and 50Mo3-2, formed distinct phylogenetic lineages within the genus *Staphylococcus* (Fig. 1). Strains Mo2-6^T^ and S9 displayed very high sequence similarity (~99.9%) to *Staphylococcus equorum* subsp. *equorum* ATCC 43958^T^ and *S. equorum* subsp. linens RP29^T^. Similarly, strains KG4-3^T^ and 50Mo3-2 were closely related to *Staphylococcus saprophyticus* subsp. saprophyticus ATCC 15305^T^ and *Staphylococcus pseudoxylosus* S04009^T^, also with high sequence similarities (~99.9%). Phylogenetic trees generated using the ML and MP algorithms further supported the clustering of strains Mo2-6^T^ and S9, as well as strains KG4-3^T^ and 50Mo3-2, within distinct *Staphylococcus* lineages (Fig. S1). Collectively, these findings suggest that strains Mo2-6^T^ and S9, along with strains KG4-3^T^ and 50Mo3-2, may represent distinct members of the genus *Staphylococcus*.

### Whole genome sequencing and genome-based phylogeny

Complete genomes for strains Mo2-6^T^, S9, KG4-3^T^, and 50Mo3-2, with sizes of approximately 2,918 kb, 2,912 kb, 3,108 kb, and 3,139 kb, respectively, were obtained by de novo assembly of MinION sequencing reads (coverages of 115×, 44×, 574×, and 53×, respectively) followed by polishing with Illumina sequencing reads (coverages of 423×, 402×, 250×, and 361×, respectively). The 16S rRNA gene sequences identified within these genomes were consistent with those obtained through PCR-based sequencing. CheckM2 analysis confirmed that all assemblies were of high quality, with 100% completeness and contamination rates of 1.5%, 0.2%, 0%, and 0.9% for strains Mo2-6^T^, S9, KG4-3^T^, and 50Mo3-2, respectively, meeting the accepted criteria for high-quality genomes (completeness ≥ 90%, contamination ≤ 10%) (Chklovski et al., 2023).

**Fig. 2.**
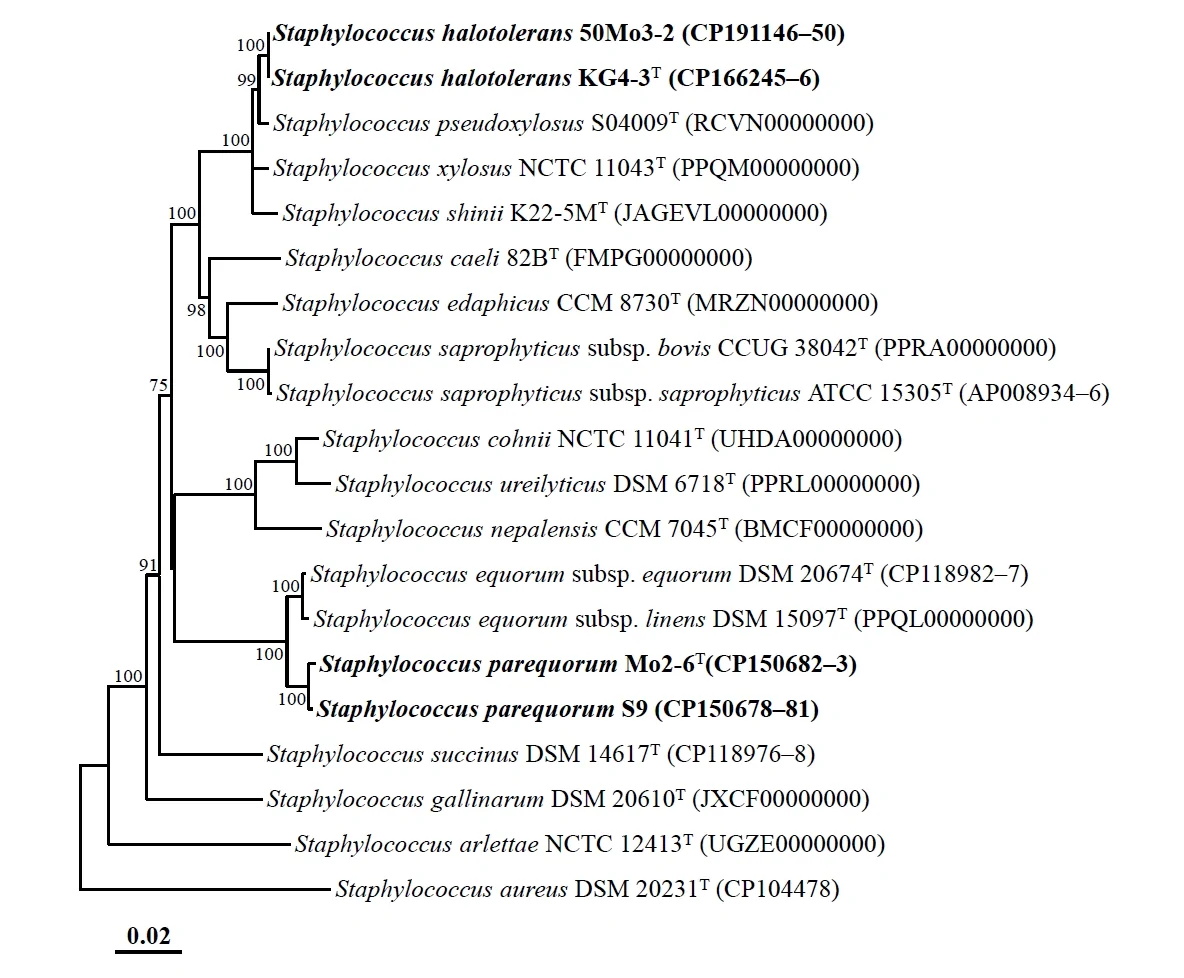
Phylogenomic tree showing the phylogenetic relationships among strains Mo2-6^T^, S9, KG4-3^T^, and 50Mo3-2, and their closely related type strains, based on the concatenated protein sequences of 120 ubiquitous single-copy marker genes (bac120 marker set). The numbers at the nodes represent bootstrap percentages from 1,000 replicates; only values greater than 70% are shown. *Staphylococcus aureus* DSM 20231^T^ (CP104478) was used as the outgroup. Scale bar: 0.02 substitutions per amino acid.

Genome-based phylogenomic analysis revealed that strains Mo2-6^T^ and S9, as well as strains KG4-3^T^ and 50Mo3-2, formed distinct phylogenetic lineages within the genus *Staphylococcus*, with 100% bootstrap support (Fig. 2), consistent with the 16S rRNA gene-based phylogeny. These findings confirm that strains Mo2-6^T^ and S9, as well as strains KG4-3^T^ and 50Mo3-2, may represent distinct members of the genus *Staphylococcus*. The ANI and dDDH values between strains Mo2-6^T^ and S9 were 99.6% and 96.3%, respectively, while those between strains KG4-3^T^ and 50Mo3-2 were 99.9% and 98.9%, respectively (Table S1). These values exceed the established species delineation thresholds (ANI ~95%, dDDH 70%) for prokaryotes (Riesco and Trujillo, 2024), indicating that strains Mo2-6^T^ and S9, as well as strains KG4-3^T^ and 50Mo3-2, belong to the same species. However, the ANI and dDDH values between strains Mo2-6^T^ and KG4-3^T^ were 80.3% and 23.9%, respectively, falling below species demarcation thresholds and suggesting that these strains represent distinct *Staphylococcus* species. Additionally, the ANI and dDDH values between strains Mo2-6^T^, S9, KG4-3^T^, and 50Mo3-2 and other *Staphylococcus* type strains were all below 95.0% and 60.1%, respectively (Table S1), indicating that these strains are distinct from previously characterized *Staphylococcus* species and likely represent novel species within the genus.

The results of the phylogenomic analysis and genome-relatedness assessments strongly suggest that strains Mo2-6^T^ and S9, as well as strains KG4-3^T^ and 50Mo3-2, represent two distinct novel species within the genus *Staphylococcus*. Based on genome-based phylogenetic analysis, *S. equorum* subsp. *equorum* KACC 13255^T^, *S. pseudoxylosus* DSM 107950^T^, and *Staphylococcus xylosus* KACC 13239^T^ were selected as reference strains for comparative analyses of genomic and phenotypic characteristics, as well as fatty acid compositions.

### Genomic features

The complete genome of strain Mo2-6^T^ consists of a single circular chromosome (2,871,546 bp, G + C content of 33.6%) and one circular plasmid (46,861 bp, G + C content of 31.0%), encoding a total of 2,874 genes, including 2,742 protein-coding sequences, 22 rRNA genes, and 61 tRNA genes. Strain S9 has a complete genome comprising a single circular chromosome (2,826,480 bp, G + C content of 33.5%) and three circular plasmids (49,048 bp, 31.0% G + C content; 32,976 bp, 30.5% G + C content; 3,741 bp, 28.0% G + C content), encoding a total of 2,852 genes, including 2,687 coding sequences, 22 rRNA genes, and 61 tRNA genes. The complete genome of strain KG4-3^T^ consists of one circular chromosome (2,971,477 bp, G + C content of 32.5%) and one circular plasmid (46,916 bp, G + C content of 29.5%), encoding 2,872 genes, including 2,763 coding sequences, 22 rRNA genes, and 61 tRNA genes. The complete genome of strain 50Mo3-2 contains a single circular chromosome (2,996,323 bp, G + C content of 33.0%) and four circular plasmids (46,917 bp, G + C content of 29.5% content; 45,438 bp, 32.0% G + C content; 45,411 bp, 33.0% G + C content; 4,884 bp, 28.5% G + C content), encoding a total of 3,106 genes, including 2,956 protein-coding sequences, 22 rRNA genes, and 85 tRNA genes. The DNA G + C contents of strains Mo2-6^T^, S9, KG4-3^T^, and 50Mo3-2, based on their entire genomes, were 33.4%, 33.3%, 32.5%, and 32.7%, respectively. The general genomic features of these strains, along with those of closely related *Staphylococcus* type strains, are summarized in Table 1. Overall, the genomic characteristics of strains Mo2-6^T^, S9, KG4-3^T^, and 50Mo3-2 are comparable to those of other *Staphylococcus* species (Table 1).

We further examined the presence of antibiotic resistance genes in the genomes of strains Mo2-6^T^, S9, KG4-3^T^, and 50Mo3-2, considering that *Staphylococcus* species often act as reservoirs for these genes, and the strains were isolated from fermented foods consumed by humans. The analysis revealed that all four strains harbor the *bla* gene, which encodes *β*-lactamase and confers resistance to *β*-lactam antibiotics (e.g., ampicillin), as well as the *fosB* gene, which encodes the AP-1 transcription factor subunit and confers resistance to fosfomycin. Additionally, strains KG4-3^T^ and 50Mo3-2 carry the *mphC* and *fusD* genes, which encode macrolide phosphotransferase and Fus family proteins, respectively, providing resistance to macrolide antibiotics and fusidic acid. In contrast, strains Mo2-6^T^ and S9 lack these additional resistance genes. Notably, only strain KG4-3^T^ carries the *erm*(44) gene, which encodes erythromycin ribosome methylase and confers resistance to erythromycin. However, the genomes of strains Mo2-6^T^, S9, KG4-3^T^, and 50Mo3-2 did not contain the *mecA* gene (found in SCCmec elements), which encodes penicillin-binding protein and provides resistance to penicillin-like antibiotics as well as methicillin (de Carvalho et al., 2024). The *str* gene, which confers resistance to streptomycin, and the *tet*(*K*) gene, which encodes a tetracycline efflux protein and provides resistance to tetracycline, were also absent. These genes are frequently found in *Staphylococcus aureus*. The profiles of antibiotic resistance genes in strains Mo2-6^T^, S9, KG4-3^T^, and 50Mo3-2 were significantly different from those in reference strains (Table 1). Although *Staphylococcus* species isolated from high-salt fermented foods exhibit resistance to various antibiotics, there is no evidence that these traits provide a selective advantage related to antimicrobial substances or salt tolerance in fermented soybean products such as ganjang. Instead, their persistence in these environments is likely due to intrinsic salt tolerance. It is more plausible that antibiotic-resistant *Staphylococcus* strains are opportunistically introduced via raw materials and participate in fermentation, rather than having specifically adapted to high-salt conditions (Han et al., 2020). Antibiotic-resistant *Staphylococcus* strains in traditional fermented soybean foods may serve as reservoirs of resistance genes, potentially transferring them to pathogenic bacteria in the human gut via food consumption. Given the high prevalence of *Staphylococcus* species in these products (Han et al., 2021; Song et al., 2021) and their broad antibiotic resistance profiles (Lee et al., 2015), *Staphylococcus* strains harboring resistance genes could pose significant food safety concern. These observations highlight the need for thorough safety assessments of *Staphylococcus* strains in fermented soybean foods and support the selection of safe strains for use as fermentation starters to reduce potential health risks.

### Phenotypic and biochemical characteristics

All strains (Mo2-6^T^, S9, KG4-3^T^, and 50Mo3-2) grew well on TSA, NA, R2A, LB agar, and MA, each containing 2% NaCl. The colonies of strains Mo2-6^T^ and S9 appeared pale white, circular, raised, and smooth with entire margins on TSA after 2 days of incubation at 30°C. In contrast, strains KG4-3^T^ and 50Mo3-2 colonies were pale white, rough, flat, and had irregular edges on TSA after 2 days of incubation at 35°C. Anaerobic growth was observed on TSA for all strains, confirming their facultatively aerobic nature. Cells of all strains were Gram-positive, non-spore-forming, non-motile, facultatively aerobic cocci, with slight size variations: approximately 0.75–0.85 µm in diameter for Mo2-6^T^, 0.6–0.7 µm for S9, 0.9–1.0 µm for KG4-3^T^, and 0.7–0.8 µm for 50Mo3-2 (Fig. S2). Most phenotypic characteristics of strains Mo2-6^T^, S9, KG4-3^T^, and 50Mo-3—including motility, oxidase, *β*-galactosidase, *β*-glucosidase, and arginine dihydrolase activities, indole production, glucose fermentation, and the ability to hydrolyze starch, esculin, casein, gelatin, L-tyrosine, Tween 20, and Tween 80—were consistent with those of closely related reference type strains (Table 2). However, certain traits, such as growth temperature, catalase activity, the assimilation of adipic acid and trisodium citrate, and urea hydrolysis, distinguished strains Mo2-6^T^ and S9, as well as KG4-3^T^ and 50Mo3-2, from other closely related *Staphylococcus* species.

Antimicrobial susceptibility testing revealed that strains Mo2-6^T^, S9, KG4-3^T^, and 50Mo3-2 were all susceptible to tetracycline, ciprofloxacin, vancomycin, chloramphenicol, rifampicin, oxacillin, penicillin, streptomycin, and kanamycin. However, differences were observed in their resistance profiles. Strains Mo2-6^T^, S9, and 50Mo3-2 were susceptible to erythromycin, while strain KG4-3^T^ exhibited resistance. In contrast, strain Mo2-6^T^ was resistant to fosfomycin, whereas the other strains were susceptible. Additionally, strain S9 was susceptible to ampicillin, unlike the other strains, which were resistant. These resistance patterns generally correlate with the presence of the corresponding antibiotic resistance genes, except for fosfomycin susceptibility in strains S9, KG4-3^T^, and 50Mo3-2, and ampicillin susceptibility in strain S9 (Table 1).

### Chemotaxonomic characteristics

MK-7 was identified as the predominant respiratory isoprenoid quinone in strains Mo2-6^T^, S9, KG4-3^T^, and 50Mo3-2, which is consistent with findings in other *Staphylococcus* strains (Kovařovic et al., 2022, 2023). Additionally, MK-6 was detected as a minor isoprenoid quinone in strains Mo2-6^T^ and S9, while MK-8 was identified as a minor isoprenoid quinone in strains KG4-3^T^ and 50Mo3-2. In terms of cellular fatty acids, anteiso-C_15:0_ and iso-C_15:0_ were the major fatty acids (comprising > 10.0% of the total fatty acids) in all strains, and their overall fatty acid profiles were similar to those of closely related *Staphylococcus* reference strains. However, notable differences in fatty acid compositions were observed (Table S2). For instance, anteiso-C_13:0_, which was identified as a minor fatty acid in other strains, was a major fatty acid in strain KG4-3^T^. Furthermore, C_16:0_ and C_18:0_, which were relatively abundant in strain KG4-3^T^, *S. equorum* subsp. *equorum* KACC 13255^T^, and *S. pseudoxylosus* DSM 107950^T^, were detected in only minor amounts in strains Mo2-6^T^, S9, and 50Mo3-2.

### Taxonomic Conclusion

The results of phylogenetic analyses based on 16S rRNA gene and genome sequences, as well as physiological and chemotaxonomic assessments, clearly demonstrate that strains Mo2-6^T^, S9, KG4-3^T^, and 50Mo3-2 belong to the genus *Staphylococcus*. However, the genome-based relatedness measures (ANI and dDDH) were below the thresholds for defining novel species. Additionally, several distinct phenotypic characteristics suggest that strains Mo2-6^T^ and S9, as well as strains KG4-3^T^ and 50Mo3-2, represent different novel species not recognized within the genus *Staphylococcus*. In conclusion, based on the phylogenetic, physiological, and chemotaxonomic evidence, we propose the names *Staphylococcus parequorum* sp. nov. for strains Mo2-6^T^ and S9, and *Staphylococcus halotolerans* sp. nov. for strains KG4-3^T^ and 50Mo3-2.

### Description of *Staphylococcus parequorum* sp. nov.

*Staphylococcus parequorum* (par.e.quo′rum. Gr. prep. *para*, next to, resembling; L. gen. masc. pl. n. *equorum*, of horses, and a specific epithet of a *Staphylococcus* species; N.L. gen. masc. n. parequorum, next to (*Staphylococcus*) *equorum*).

Colonies grown on TSA with 2% NaCl are pale white, circular, raised, smooth, and have entire margins. Cells are Gram-positive, facultatively aerobic, non-spore-forming, coagulase-negative, non-motile cocci, and exhibit negative oxidase and catalase activities. Growth occurs between 4°C and 40°C (optimum, 30°C), within a pH range of 6.0–10.0 (up to 9.0 depending on the strain, with an optimum at pH 7.0), and in the presence of 0–22% (w/v) NaCl (optimum, 2%). Hydrolysis of casein, esculin, and urea is positive, while starch, gelatin, L-tyrosine, Tween 20, and Tween 80 hydrolysis is negative. Nitrate is reduced to nitrite, but no nitrogen gas is produced. Indole production and D-glucose fermentation are negative. The strain shows positive activities for *β*-glucosidase and *β*-galactosidase. Assimilation of D-glucose, *N*-acetyl-glucosamine, D-maltose, L-arabinose, D-mannose, D-mannitol, malic acid, and potassium gluconate is positive, while assimilation of capric acid, phenylacetic acid, and trisodium citrate is negative. Assimilation of adipic acid is variable. MK-7 is identified as the major isoprenoid quinone, with MK-6 detected as a minor component. The major cellular fatty acids (> 10%) are iso-C_15:0_ and anteiso-C_15:0_.

The type strain is Mo2-6^T^ (=KACC 23865^T^ =JCM 37038^T^), isolated from a traditional Korean soybean food. The DNA G + C content of the type strain is 33.4% (calculated from the whole genome sequence). The GenBank accession numbers for the 16S rRNA gene and genome sequences of strain Mo2-6^T^ are PP524727 and CP150682-3, respectively.

### Description of *Staphylococcus halotolerans* sp. nov.

*Staphylococcus halotolerans* (ha.lo.to′le.rans. Gr. masc. n. *hals*, salt; L. part. *tolerans*, tolerating; N.L. part. adj. *halotolerans*, referring to the ability to tolerate high salt concentrations).

Colonies grown on TSA with 2% NaCl are pale white, rough in texture, flat, and have irregular edges. Cells are Gram-positive, facultatively aerobic, non-spore-forming, coagulase-negative, non-motile cocci, exhibiting catalase-positive and oxidase-negative activities. Growth occurs between 4°C and 40°C (optimum at 35°C), within a pH range of 6.0–10.0 (optimum at pH 7.0), and in the presence of 0–28% (w/v) NaCl (optimum at 2%). Hydrolysis of casein, esculin, and urea is positive, while hydrolysis of starch, gelatin, L-tyrosine, Tween 20, and Tween 80 is negative. Nitrate is reduced to nitrite, but no nitrogen gas is produced. Indole production and D-glucose fermentation are negative. The strain shows positive activities for *β*-glucosidase and *β*-galactosidase. Assimilation of D-glucose, *N*-acetyl-glucosamine, D-maltose, L-arabinose, D-mannose, D-mannitol, malic acid, potassium gluconate, and trisodium citrate is positive, while assimilation of adipic acid, capric acid, and phenylacetic acid is negative. MK-7 is identified as the major isoprenoid quinone, with MK-8 detected as a minor component. The major cellular fatty acids (> 10%) are iso-C_15:0_ and anteiso-C_15:0_, with anteiso-C_13:0_ also being a major fatty acid in certain strains.

The type strain is KG4-3^T^ (=KACC 23684^T^ =JCM 37037^T^), isolated from a traditional Korean soybean food. The DNA G + C content of the type strain is 32.5% (calculated from the whole genome sequence). The GenBank accession numbers for the 16S rRNA gene and genome sequences of strain KG4-3^T^ are PP524725 and CP166245–6, respectively.

## Figures and Tables

**Table 1.** General genomic features of strains Mo2-6^T^, S9, 50Mo3-2^T^, and KG4-3 and their closely related *Staphylococcus* type strains Taxa: 1, strain Mo2-6^T^ (CP150682–3); 2, strain S9 (CP150678–81); 3, strain KG4-3^T^ (CP166245–6); 4, strain 50Mo3-2 (CP191146–50); 5, *S. equorum* subsp. *equorum* DSM 20674^T^ (CP118982–7); 6, *S. pseudoxylosus* S04009^T^ (RCVN00000000); 7, *S. xylosus* NCTC 11043^T^ (PPQM00000000)

Characteristic^*^	1	2	3	4	5	6	7
Genome status^†^ (no. of contigs)	C (2)	C (4)	C (2)	C (5)	C (6)	D (124)	D (2)
Genome size (kb)	2,918	2,912	3,108	3,139	2,741	3,046	2,726
G + C contents (%)	33.4	33.3	32.5	32.7	33.1	32.8	32.9
No. of total genes	2,874	2,852	2,872	3,106	2,664	3,013	2,615
No. of protein-coding genes	2,742	2,687	2,763	2,956	2,548	2,898	2,515
No. of total RNA genes	87	87	87	111	86	51	71
No. of tRNA genes	61	61	61	85	60	42	60
No. of rRNAs (5S, 16S, and 23S)	8, 7, 7	8, 7, 7	8, 7, 7	8, 7, 7	8, 7, 7	1, 2, 2	3, 1, 3
Presence of antibiotic resistance genes^‡^							
*bla*	+	+	+	+	+	+	+
*mecA*	–	–	–	–	–	+	–
*erm*(44)	–	–	+	–	–	–	–
*mphC*	–	–	+	+	+	+	–
*fosB*	+	+	+	+	–	+	+
*fusD*	–	–	+	+	–	+	+
*str*	–	–	–	–	+	–	–
*tet(K)*	–	–	–	–	+	–	–

* The information was obtained from the GenBank database and annotated using the NCBI prokaryotic genome annotation pipeline (www.ncbi.nlm.nih.gov/genome/annotation_prok/).

† C, complete; D, draft

‡ *bla*, *β*-lactamase; *mecA*, penicillin-binding protein; *erm*(44), erythromycin ribosome methylase; *mphC*, macrolide phosphotransferase; *fosB*, transcription factor AP-1 subunit; *fusD*, Fus family protein; *str*, streptomycin-resistant gene; *tet*(*K*), tetracycline efflux protein

**Table 2.** Differential phenotypic characteristics of strains Mo2-6^T^, S9, 50Mo3-2^T^, and KG4-3 and their closely related *Staphylococcus* type strains Taxa: 1, strain Mo2-6^T^ (this study); 2, strain S9 (this study); 3, strain KG4-3^T^ (this study); 4. strain 50Mo3-2 (this study); 5, *S. equorum* subsp. *equorum* KACC 13255^T^ (Schleifer et al., 1984); 6, *S. pseudoxylosus* DSM 107950^T^ (MacFadyen et al., 2019); 7, *S. xylosus* KACC 13239^T^ (Schleifer et al., 1975). All strains are facultatively anaerobic, non-motile, coagulase-negative cocci, and are positive for the following characteristics: hydrolysis* of esculin and casein, NO_3_^-^ reduction to NO_2_^-^*, enzyme activity* of *β*-glucosidase and *β*-galactosidase, and assimilation* of D-glucose, *N*-acetyl-glucosamine, D-maltose, and potassium gluconate. All strains are negative for the following characteristics: indole production, glucose fermentation, hydrolysis* of starch, gelatin, L-tyrosine, Tween 20, and Tween 80, enzyme activity* of oxidase and arginine dihydrolase, and assimilation* of capric acid and phenylacetic acid. Symbols: +, positive; –, negative; *w*, weakly positive

Characteristic	1	2	3	4	5	6	7
Isolation source	Ganjang	Ganjang	Meju	Ganjang	Horse skin	Bovine mastitis	Human skin
Colony color	Pale white	Pale white	Pale white	Pale white	Pale white	white	Light yellow
Growth range^*^ of:							
Temperature (°C, optimum)	4–40 (30)	4–40 (30)	4–40 (35)	4–40 (35)	10–40 (30)	10–40 (35)	10–40 (35)
NaCl (%, optimum)	0–22 (2)	0–22 (2)	0–28 (2)	0–28 (2)	0–20 (2)	0–20 (0–2)	0–22 (0)
pH (optimum)	6–10 (7)	6–9 (7)	6–10 (7)	6–10 (7)	6–11 (7)	5–10 (7-8)	6–11 (8)
Hydrolysis^*^ of:							
Urea	+	+	+	+	+	–	+
Assimilation^*^ of:							
L-Arabinose	+	*w*	+	+	+	+	+
D-Mannose	*+*	*+*	*+*	*+*	*w*	*+*	*+*
D-Mannitol	+	*w*	+	+	+	+	+
Adipic acid	–	+	–	–	–	–	–
Malic acid	*+*	*w*	+	+	+	+	+
Trisodium citrate	–	–	*+*	+	+	+	+
Enzyme activity^*^ of:							
Catalase	–	–	+	+	–	+	+

* These analyses were conducted under the same conditions in this study.

## Data Availability

The GenBank/EMBL/DDBJ accession numbers for the 16S rRNA genes and genome sequences of strains Mo2-6^T^, S9, KG4-3^T^, and 50Mo3-2 are PP524727 and CP150682–3, PP524728 and CP150678–81, PP524725 and CP166245–6, and PP524726 and CP191146–50, respectively.

## Supplementary Information

Table S1. Genome relatedness among strains Mo2-6^T^, S9, and KG4-3^T^ and 50Mo3-2 and their closely related *Staphylococcus* type strains Taxa: 1, strain Mo2-6^T^ (CP150682–3); 2, strain S9 (CP150678–81); 3, strain KG4-3^T^ (CP166245–6); 4. strain 50Mo3-2 (CP191146–50); 5, *S. equorum* subsp. *equorum* DSM 20674^T^ (CP118982–7); 6, *S. pseudoxylosus* S04009^T^ (RCVN00000000); 7, *S. xylosus* NCTC 11043^T^ (UHEI00000000)

Table S2. Cellular fatty acid compositions (%) of strains Mo2-6^T^, S9, KG4-3^T^, and 50Mo3-2 and their closely related *Staphylococcus* type strains Taxa: 1, strain Mo2-6^T^; 2, strain S9; 3, strain KG4-3^T^; 4, strain 50Mo3-2; 5, *S. equorum* subsp. *equorum* KACC 13255^T^; 6, *S. pseudoxylosus* DSM 107950^T^; 7, *S. xylosus* KACC 13239^T^. All data were obtained from this study. Data are expressed as percentages of the total fatty acids, and fatty acids constituting less than 1.0% in all strains are not shown. Major components (> 10.0%) are highlighted in bold; symbols: tr, trace amount (< 1.0%); –, not detected

Fig. S1. Maximum-likelihood (A) and maximum-parsimony (B) trees showing the phylogenetic relationships among strains Mo2-6^T^, S9, KG4-3^T^, and 50Mo3-2, and their closely related type strains, based on 16S rRNA gene sequences. Numbers at the nodes represent bootstrap percentages from 1,000 replicates; only values greater than 70% are shown. *Staphylococcus aureus* ATCC 12600^T^ (X68417) was used as the outgroup. Scale bars in panels A and B represent substitutions per nucleotide and nucleotide substitutions over the entire sequences, respectively.

Fig. S2. Transmission electron micrographs of negatively stained cells (using 2% (w/v) uranyl acetate) showing the general cellular morphologies of strains Mo2-6^T^ (A), S9 (B), KG4-3^T^ (C), and 50Mo3-2 (D) cultivated on tryptic soy agar at 30°C for 2 days. Scale bars, 500 nm.
