# Corrigendum: *Staphylococcus parequorum* sp. nov. and *Staphylococcus halotolerans* sp. nov., isolated from traditional Korean soybean foods

**DOI:** 10.71150/jm.2510101

**Published:** 2025-10-23

**Authors:** Ju Hye Baek, Dong Min Han, Dae Gyu Choi, Chae Yeong Moon, Jae Kyeong Lee, Chul-Hong Kim, Jung-Woong Kim, Che Ok Jeon

**Affiliations:** Department of Life Science, Chung-Ang University, Seoul 06974, Republic of Korea

**Corrigendum: Journal of Microbiogy. 2025. 63: e2503003.**

https://doi.org/10.71150/jm.2503003

­

The original online version of this article was revised:

This article contained an error in the strain deposit number of the type strain in the Results and Discussion section.

The corrected protologue is provided below.

## Results and Discussion

### Description of *Staphylococcus parequorum* sp. nov.

­

**The originally published version read as follows:**

The type strain is Mo2-6^T^ (=KACC 23865^T^ =JCM 37038^T^), isolated from a traditional Korean soybean food.

­

**It should be read as follows:**

The type strain is Mo2-6^T^ (=KACC 23685^T^ =JCM 37038^T^), isolated from a traditional Korean soybean food.

­

**The corrected protologue is provided below:**

### Description of *Staphylococcus parequorum* sp. nov.

*Staphylococcus parequorum* (par.e.quo′rum. Gr. prep. *para*, next to, resembling; L. gen. masc. pl. n. *equorum*, of horses, and a specific epithet of a *Staphylococcus* species; N.L. gen. masc. n. *parequorum*, next to (*Staphylococcus*) *equorum*).

Colonies grown on TSA with 2% NaCl are pale white, circular, raised, smooth, and have entire margins. Cells are Gram-positive, facultatively aerobic, non-spore-forming, coagulase-negative, non-motile cocci, and exhibit negative oxidase and catalase activities. Growth occurs between 4°C and 40°C (optimum, 30°C), within a pH range of 6.0–10.0 (up to 9.0 depending on the strain, with an optimum at pH 7.0), and in the presence of 0%–22% (w/v) NaCl (optimum, 2%). Hydrolysis of casein, esculin, and urea is positive, while starch, gelatin, ʟ-tyrosine, Tween 20, and Tween 80 hydrolysis is negative. Nitrate is reduced to nitrite, but no nitrogen gas is produced. Indole production and ᴅ-glucose fermentation are negative. The strain shows positive activities for *β*-glucosidase and *β*-galactosidase. Assimilation of ᴅ-glucose, *N*-acetyl-glucosamine, ᴅ-maltose, ʟ-arabinose, ᴅ-mannose, ᴅ-mannitol, malic acid, and potassium gluconate is positive, while assimilation of capric acid, phenylacetic acid, and trisodium citrate is negative. Assimilation of adipic acid is variable. MK-7 is identified as the major isoprenoid quinone, with MK-6 detected as a minor component. The major cellular fatty acids (> 10%) are iso-C_15:0_ and anteiso-C_15:0_.

The type strain is Mo2-6^T^ (=KACC 23685^T^ =JCM 37038^T^), isolated from a traditional Korean soybean food. The DNA G + C content of the type strain is 33.4% (calculated from the whole genome sequence). The GenBank accession numbers for the 16S rRNA gene and genome sequences of strain Mo2-6^T^ are PP524727 and CP150682–3, respectively.

