# Supplementary material for: Staphylococcus parequorum sp. nov. and Staphylococcus halotolerans sp. nov., isolated from traditional Korean soybean foods
Source: J Microbiol. 2025 Aug 31;63(8):e2503003. doi: 10.71150/jm.2503003 (PMC13577055; doi:10.71150/jm.2503003)

Fig. S1. Maximum-likelihood (A) and maximum-parsimony (B) trees showing the phylogenetic relationships among strains Mo2-6^{T}, S9, KG4-3^{T}, and 50Mo3-2, and their closely related type strains, based on 16S rRNA gene sequences. Numbers at the nodes represent bootstrap percentages from 1,000 replicates; only values greater than 70% are shown. *Staphylococcus aureus* ATCC 12600^{T} (X68417) was used as the outgroup. Scale bars in panels A and B represent substitutions per nucleotide and nucleotide substitutions over the entire sequences, respectively.

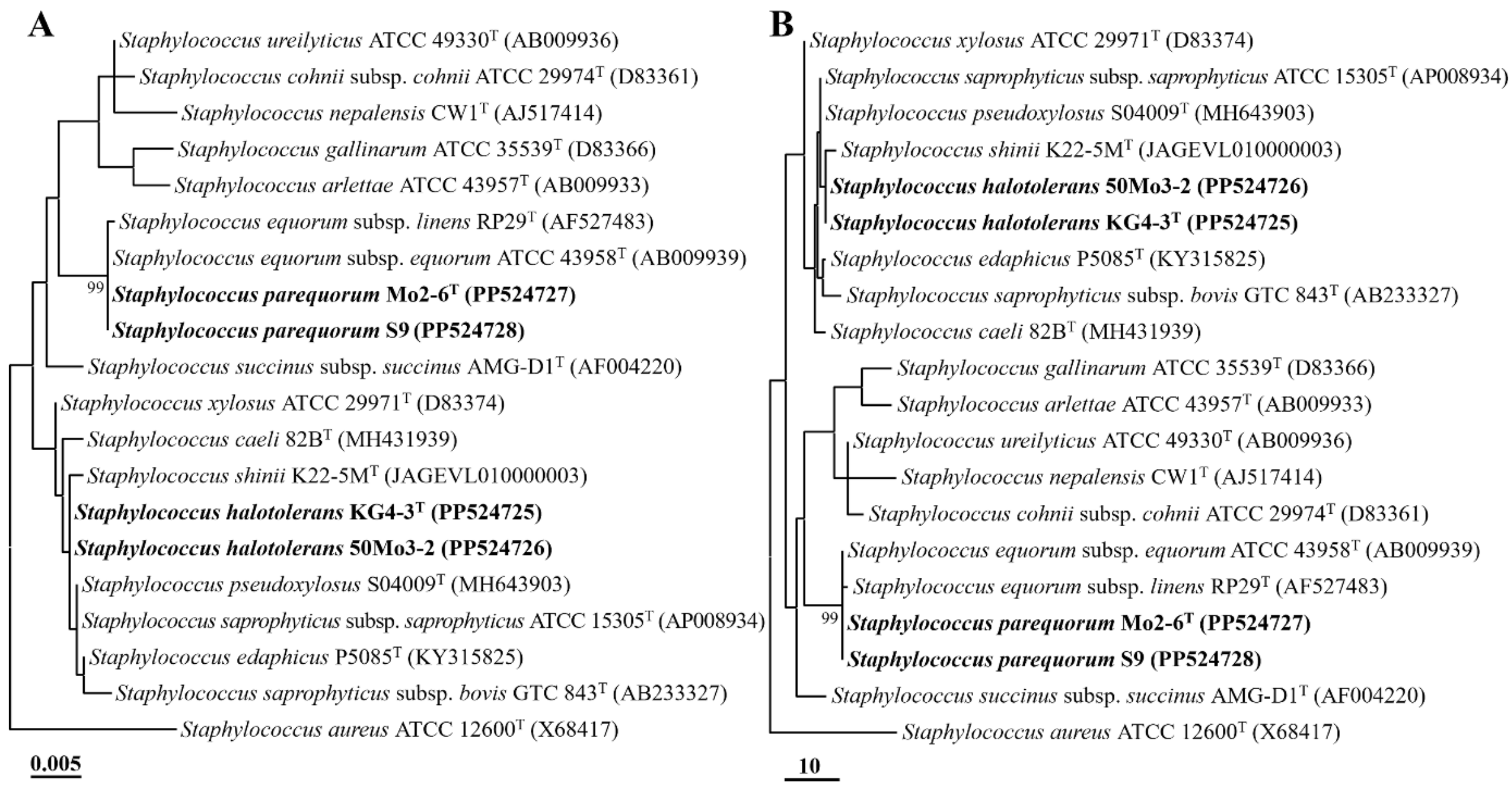

Supplement: Supplementary file 1 [file jm-2503003-Supplementary-Fig-S1.pdf]
