# Supplementary material for: Staphylococcus parequorum sp. nov. and Staphylococcus halotolerans sp. nov., isolated from traditional Korean soybean foods
Source: J Microbiol. 2025 Aug 31;63(8):e2503003. doi: 10.71150/jm.2503003 (PMC13577055; doi:10.71150/jm.2503003)

Fig. S2. Transmission electron micrographs of negatively stained cells (using 2% (w/v) uranyl acetate) showing the general cellular morphologies of strains Mo2-6^T (A), S9 (B), KG4-3^T (C), and 50Mo3-2 (D) cultivated on tryptic soy agar at 30°C for 2 days. Scale bars, 500 nm.

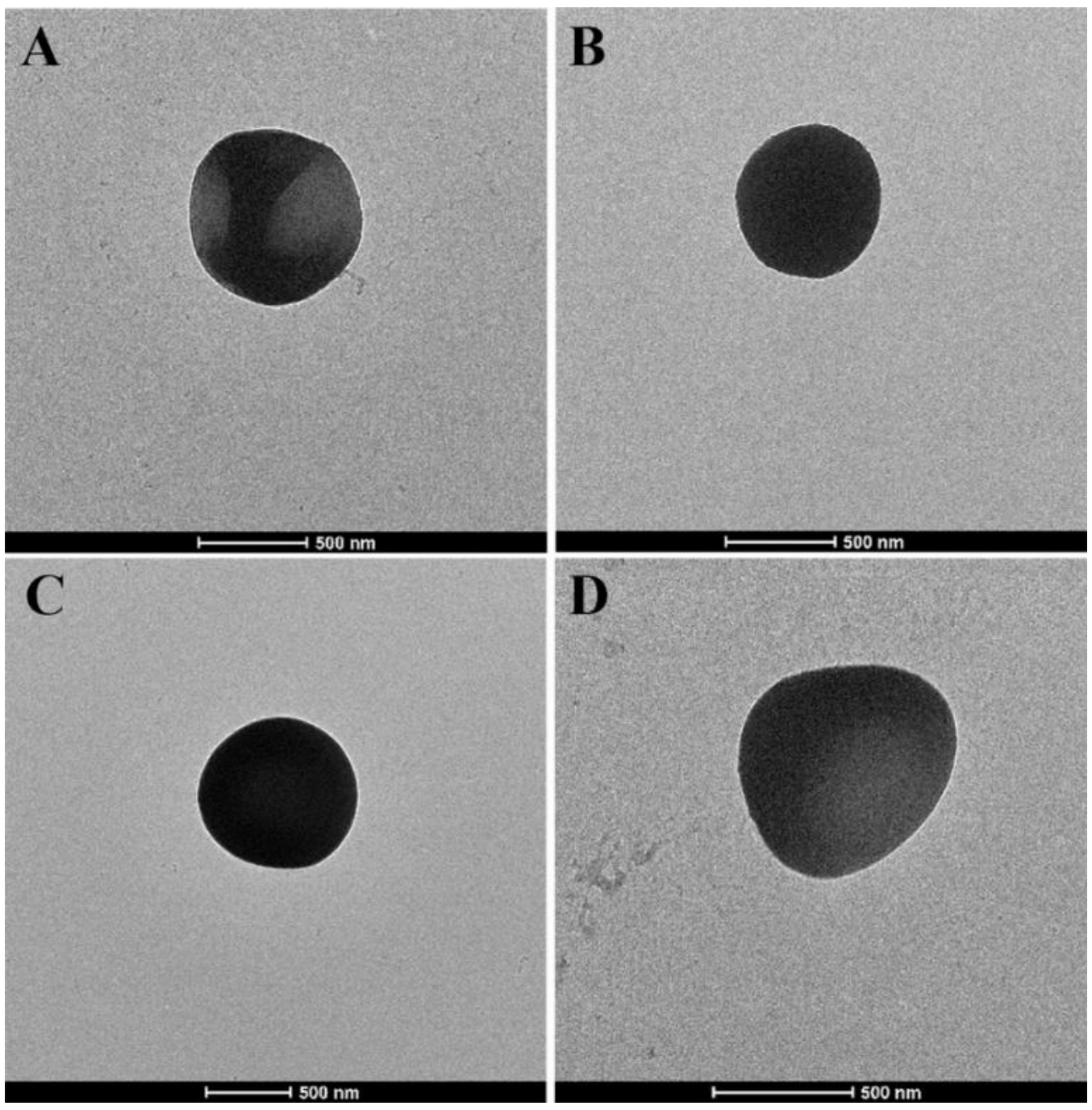

Supplement: Supplementary file 2 [file jm-2503003-Supplementary-Fig-S2.pdf]
