# Supplementary material for: Staphylococcus parequorum sp. nov. and Staphylococcus halotolerans sp. nov., isolated from traditional Korean soybean foods
Source: J Microbiol. 2025 Aug 31;63(8):e2503003. doi: 10.71150/jm.2503003 (PMC13577055; doi:10.71150/jm.2503003)
Supplement: Supplementary file 3 [file jm-2503003-Supplementary-Table-S1.pdf]

Table S1. Genome relatedness among strains Mo2-6^T, S9, and KG4-3^T and 50Mo3-2 and their closely related *Staphylococcus* type strains

Taxa: 1, strain Mo2-6^T (CP150682–3); 2, strain S9 (CP150678–81); 3, strain KG4-3^T (CP166245–6); 4. strain 50Mo3-2 (CP191146–50); 5, *S. equorum* subsp. *equorum* DSM 20674^T (CP118982–7); 6, *S. pseudoxylosus* S04009^T (RCVN00000000); 7, *S. xylosus* NCTC 11043^T (UHEI00000000)

		dDDH† value (%)						
		**1**	**2**	**3**	**4**	**5**	**6**	**7**
ANI† value (%)	**1**	–	96.3	23.9	24.2	60.1	23.7	23.9
	**2**	99.6	–	23.9	23.8	59.8	23.7	23.9
	**3**	80.3	80.2	–	98.9	23.8	59.8	50.5
	**4**	80.3	80.2	99.9	–	23.8	59.6	50.5
	**5**	95.0	94.9	80.1	80.0	–	23.7	23.9
	**6**	80.0	80.0	94.8	94.9	80.0	–	50.6
	**7**	80.1	80.0	93.1	93.1	80.2	93.2	–

†ANI, average nucleotide identity; dDDH, digital DNA–DNA hybridization
