# Supplementary material for: Staphylococcus parequorum sp. nov. and Staphylococcus halotolerans sp. nov., isolated from traditional Korean soybean foods
Source: J Microbiol. 2025 Aug 31;63(8):e2503003. doi: 10.71150/jm.2503003 (PMC13577055; doi:10.71150/jm.2503003)
Supplement: Supplementary file 4 [file jm-2503003-Supplementary-Table-S2.pdf]

Table S2. Cellular fatty acid compositions (%) of strains Mo2-6^T, S9, KG4-3^T, and 50Mo3-2 and their closely related *Staphylococcus* type strains

Taxa: 1, strain Mo2-6^T; 2, strain S9; 3, strain KG4-3^T; 4, strain 50Mo3-2; 5, *S. equorum* subsp. *equorum* KACC 13255^T; 6, *S. pseudoxylosus* DSM 107950^T; 7, *S. xylosus* KACC 13239^T. All data were obtained from this study. Data are expressed as percentages of the total fatty acids, and fatty acids constituting less than 1.0% in all strains are not shown. Major components (> 10.0%) are highlighted in bold; symbols: tr, trace amount (< 1.0%); –, not detected

**Fatty acid**	**1**	**2**	**3**	**4**	**5**	**6**	**7**
Saturated:							
$C_{14:0}$	1.1	1.2	2.6	1.2	1.9	2.4	tr
$C_{16:0}$	3.4	3.6	9.9	2.1	**15.0**	9.2	1.8
$C_{18:0}$	2.0	2.5	7.3	2.6	**12.5**	7.2	2.2
$C_{20:0}$	tr	tr	tr	2.1	tr	tr	1.4
Unsaturated:							
iso-$C_{17:1}$ $\omega 10c$	1.4	tr	–	tr	–	–	–
$C_{18:1}$ $\omega 9c$	tr	tr	tr	tr	1.2	tr	tr
Branched:							
iso-$C_{13:0}$	2.1	1.0	9.9	6.7	1.7	6.9	tr
iso-$C_{14:0}$	1.0	tr	1.2	1.2	tr	tr	1.0
iso-$C_{15:0}$	**23.6**	**19.2**	**13.8**	**18.9**	**11.5**	**10.7**	**15.4**
iso-$C_{16:0}$	tr	tr	1.6	1.7	tr	1.4	1.4
iso-$C_{17:0}$	1.1	1.1	4.1	6.5	2.0	6.2	5.2
iso-$C_{19:0}$	tr	tr	1.1	1.8	tr	1.1	1.5
anteiso-$C_{11:0}$	1.6	–	tr	tr	–	tr	–
anteiso-$C_{13:0}$	2.1	tr	**10.8**	7.1	tr	8.9	tr
anteiso-$C_{15:0}$	**53.6**	**61.6**	**25.2**	**35.9**	**41.9**	**27.7**	**56.5**
anteiso-$C_{17:0}$	1.1	1.5	4.7	6.8	3.1	9.3	9.0
Summed feature*:							
8 ($C_{18:1}$ $\omega 6c$ and/or $C_{18:1}$ $\omega 7c$)	tr	tr	tr	tr	1.8	1.9	–

*Summed feature is a group of typically two or three fatty acids that cannot be reliably separated from one another using the selected chromatographic conditions. The MIDI system combines these fatty acids into a single feature and assigns a single percentage of the total.
